# Uric Acid Promotes Apoptosis in Human Proximal Tubule Cells by Oxidative Stress and the Activation of NADPH Oxidase NOX 4

**DOI:** 10.1371/journal.pone.0115210

**Published:** 2014-12-16

**Authors:** Daniela Verzola, Elena Ratto, Barbara Villaggio, Emanuele Luigi Parodi, Roberto Pontremoli, Giacomo Garibotto, Francesca Viazzi

**Affiliations:** 1 University of Genoa and I.R.C.C.S. Azienda Ospedaliera Universitaria San Martino-IST, Istituto Nazionale per la Ricerca sul Cancro, Department of Internal Medicine, Genoa, Italy; 2 I.R.C.C.S. Azienda Ospedaliera Universitaria San Martino-IST, Istituto Nazionale per la Ricerca sul Cancro, Clinica Nefrologica, Dialisi e Trapianto, Genoa, Italy; National Institutes of Health, United States of America

## Abstract

Mild hyperuricemia has been linked to the development and progression of tubulointerstitial renal damage. However the mechanisms by which uric acid may cause these effects are poorly explored. We investigated the effect of uric acid on apoptosis and the underlying mechanisms in a human proximal tubule cell line (HK-2). Increased uric acid concentration decreased tubule cell viability and increased apoptotic cells in a dose dependent manner (up to a 7-fold increase, p<0.0001). Uric acid up-regulated Bax (+60% with respect to Ctrl; p<0.05) and down regulated X-linked inhibitor of apoptosis protein. Apoptosis was blunted by Caspase-9 but not Caspase-8 inhibition. Uric acid induced changes in the mitochondrial membrane, elevations in reactive oxygen species and a pronounced up-regulation of NOX 4 mRNA and protein (p<0.05). In addition, both reactive oxygen species production and apoptosis was prevented by the NADPH oxidase inhibitor DPI as well as by Nox 4 knockdown. URAT 1 transport inhibition by probenecid and losartan and its knock down by specific siRNA, blunted apoptosis, suggesting a URAT 1 dependent cell death. In summary, our data show that uric acid increases the permissiveness of proximal tubule kidney cells to apoptosis by triggering a pathway involving NADPH oxidase signalling and URAT 1 transport. These results might explain the chronic tubulointerstitial damage observed in hyperuricaemic states and suggest that uric acid transport in tubular cells is necessary for urate-induced effects.

## Introduction

Although unproven in humans, an emergent hypothesis is that hyperuricemia has a causative effect on the development [1]–[4] and progression of tubulointerstitial chronic renal damage in several clinical conditions, such as hypertension, diabetes, and chronic glomerulonephritis [5]–[9].

The mechanisms by which uric acid (UA) leads to tissue damage and contributes to the development of tubulointerstitial damage are not understood. Recent observations indicate that UA triggers the generation of free radicals and oxidant stress in several cell types [10]–[11]. Reactive oxygen species (ROS) are considered to be important mediators for several biologic responses, including proliferation, extracellular matrix deposition, and apoptosis [12]. How UA contributes to oxidative stress is still a matter of debate [13]. On one hand urate contributes about 60% of plasma antioxidant activity, it is a free radical scavenger that is effective against peroxynitrite, superoxide, hydroxyl ions, and singlet oxygen as well as reducing oxidant formation by chelating iron [14]. On the other hand the observed UA induced effects include a number of different pathogenetic mechanisms in different cells and tissues, such as the activation of critical proinflammatory pathways [15]–[16] and stimulation of cell proliferation in VSMC [17], the decrease in NO bioavailability and cell proliferation in endothelial cells [18]–[19].

Recently, urate induced oxidative and inflammatory damage have been described in mouse differentiated adipocytes [11]. Moreover, urate has been shown to raise systemic blood pressure by activating the renin-angiotensin system and by promoting sodium sensitivity in animal models [20]. In the kidney, UA has been shown to induce MCP-1 production [21] and MAPK signaling pathway in human kidney proximal tubular cells (HuPTCs) [22]. In addition, UA associated danger signals have been proposed to activate the NALP3 inflammasome [23] with subsequent secretion of IL-1β and IL-18 and further pro-inflammatory events in rats with streptozocin-induced diabetes [24].

More recently, it has been suggested that elevated UA might induce renal tubule cell apoptosis by inducing an imbalance between anti-apoptotic and pro-apoptotic proteins [22]. Apoptosis, a mode of cell death mediated by the activation of caspases and characterized by cleavage of protein substrates and DNA fragmentation, is one of the major mechanism contributing to kidney cell loss and tubular atrophy in chronic kidney diseases [25]–[26]. In addition, tubular cell apoptosis has also been reported in renal biopsy of patients with familial gouty nephropathy [27].

Here, we examine the role and mechanisms of UA on apoptosis and the effects of inhibition of UA transport in HuPTCs. Our results demonstrate that UA promote apoptosis of tubular cells through a NADPH oxidase-dependent mechanism and activation of intrinsic apoptotic pathway. All these effects are prevented by the inhibition/knock down of URAT-1 strongly suggesting intracellular transport of urate is a prerequisite for UA induced cytopathic events in proximal tubular cells.

## Materials and Methods

### Cell culture

HK-2 cells, an immortalized proximal tubular epithelial cell line from normal adult human male kidney, were obtained from ATCC. Cells were grown in DMEM/F12 medium supplemented with 5% [v/v] FBS, 100 U/ml penicillin-streptomycin, 2 mmol L-glutamine, 5 µg/ml insulin, 5 µg/ml transferrin, 5 ng/ml sodium selenite, 5 pg/ml T3, 5 ng/ml hydrocortisone, 5 pg/ml PGE1 and 10 ng/ml epidermal growth factor. Cells were grown at 37°C in a humidified 5% CO_2_ condition.

### Cell treatments

HK-2 were grown to subconfluence in normal growth medium and apoptotic damage was induced by additives and serum deprivation for 48 hours in the presence of UA (Sigma Chemical Company, St. Louis, MO, USA) (7.5–12 mg/dl). The effects of UA on cell viability and apoptotic damage were studied by testing different UA concentrations (7.5–12 mg/dl) for 48 hours. Control cells (Ctrl) were not treated with UA. In order to determine whether UA is directly involved in apoptosis, Probenecid (20 µM) and Losartan (1–10 µM), were added 1 hour earlier than 12 mg/dl UA. To determine a role of caspases in 12 mg/dl UA-induced cells death, HK-2 cells were pre-incubated for 30 minutes with 50 µmol/L caspase- 8[Z-IEDT-FMK] -9[Z-LEHD-FMK] inhibitors. In order to understand if UA is directly involved in mitochondrial apoptotic pathway, we examined the effects of 12 mg/dl UA on mitochondrial transmembrane potential. The role of oxidative stress on UA apoptosis was studied by evaluating NOX 4 subunit mRNA overexpression and by preincubating cells for 30 min with 10 µM diphenylene iodonium (DPI) (Sigma Chemical Company). In addition, we tested the effects of Nox 4 silencing and anti-oxidant N-acetyl-cysteine (NAC) on UA-induced apoptosis.

### MTT assay

This assay for cell viability is based on the reduction of 3-(4.5-dimethylthiazol-2-yl)-2.5-diphenyltetrazolium bromide (MTT) (Sigma Chemical Company) by mitochondrial dehydrogenase in viable cells to produce a purple formazan product. Each experiment was performed according to the protocol as previously described [28].

### RNA interference

HK-2 were transfected with 60 nM URAT 1 and Nox 4 specific siRNAs or negative control siRNA (Ambion, Carlsbad, CA) using Lipofectamine (Invitrogen, Carlsbad, CA) according to the manufacturer’s protocol and then incubated at 37°C in a CO2 incubator for 24 hours until the cells were ready for assay. The efficacy of knockdown was determined by real-time PCR.

### MitoCapture Assay

HK-2 cells were exposed for 48 hours to 12 mg/dl UA and then, were incubated with MitoCapture Mitochondrial apoptosis detection kit (Biovision, CA 94043 U.S.A.). Disruption of mitochondrial transmembrane potential is one of the earliest intracellular events that occurs following induction of apoptosis. The kit utilizes MitoCapture, a cationic dye that fluoresces differently in healthy and in apoptotic cells. In healthy cells it aggregates in the mitochondria, giving off a bright red fluorescence. In apoptotic cells, MitoCapture cannot aggregate in the mitochondria due to the altered mitochondrial transmembrane potential, and thus it remains in the cytoplasm in its monomer form, fluorescing green. The cells were observed under fluorescence microscope and we determined the percentage of HK-2 that lose mitochondrial integrity.

### Cleaved caspase-3 evaluation

HK-2 grown on chamber slides to sub-confluence were incubated for 48 hrs with or without UA (12 mg/dl). After a five minute incubation in cold methanol, cells were incubated with anti-cleaved caspase-3 (Asp175) rabbit mAb (Cell Signaling Technology, MA, USA). Immunostaining was performed as described previously. Slides were counterstained with haematoxylin and examined by light microscopy. Apoptotic cells were expressed as % cleaved caspase 3 positive respect to total cells counted (300 cells for each condition and experiment).

### Annexin V-FITC/propidium iodide (PI) staining

In multiple experiments, HK-2 cells were grown on chamber slides, stained with FITC annexin V/propidium iodide (MBL, MA, U.S.A.). Cells were observed and counted (∼300 cells for each condition) under a fluorescence microscope using dual filter set for FITC and rhodamine. Cells that lost membrane integrity showed red staining throughout the nucleus and a halo of green staining on the cell surface. To evaluate apoptotic phenomena, we considered the percentage of cells annexin V–positive/propidium iodide–negative respect to total cells counted.

### Western blot analysis

The cell layers were lysed in cold buffer (20 mM HEPES, 150 mM NaCl, 10% [v/v] glycerol, 0.5% [v/v] NP-40, 1 mM EDTA, 2.5 mM DTT, 10 µg/L aprotinin, leupeptin, pepstatin A, 1 mM PMSF, and Na_3_VO_4_). Protein concentration was determined by using the Bicinchonic Protein assay kit (Euroclone S.p.A., Milan Italy) and 20 µg were resolved on SDS-polyacrylamide gels and electro-transferred to a PVDF membrane (Millipore Billerica, MA, U.S.A.). Blots were probed using antihuman BAX (Santa Cruz Biotechnology, Santa Cruz, CA, USA), antihuman XIAP (MBL, MA, USA), anti Nox 4 (Santa Cruz Biotechnology) and incubated in horseradish peroxidase secondary antibodies (Cell Signaling Technology), Immunoblots were developed with Immobilon Western chemiluminescent HRP substrate (Millipore Billerica, MA, U.S.A.). Band intensities were determined using Alliance imaging system (Uvitec, Cambridge, U.K.).

### Measurement of reactive oxygen species (ROS) production

HK-2 cells were incubated with 20 µM 2′–7′ dichlorofluorescein-diacetate (DCFH-DA) (Sigma Chemical Company), a cell-permeable, nonfluorescent precursor of DCF. This non-fluorescent product accumulates in the cells and the subsequent oxidation by H_2_O_2_, Fenton-type species and hydroxyl radical yields the highly fluorescent DCF product. Observations were made with a Leica DMLB microscope (Leica microsystems AG, Wetzlar, Germany). To confirm oxidative stress, HK-2 were tested with Hydroethidine (HE) (Polysciences, Inc, PA, U.S.A.), which is oxidized by ROS to become ethidium, which intercalates into the DNA and emits a red color. Cells were exposed to HE for 15 min at room temperature. For both stainings, cells were analyzed on a fluorescence microscope.

### mRNA Analysis

HK-2 were incubated for 5 hours with or without 12 mg/dl UA and total RNA was isolated using the Qiazol Lysis reagent (Qiagen Sciences, Maryland, USA). RNA sample was treated with DNase and the cleanup of the RNA was performed using an RNeasy kit (Qiagen Sciences). The RNA concentration and integrity were evaluated on a NanoDrop ND-1000 Spectrophotometer (NanoDrop Technologies Inc., Wilmington, DE, USA). 1 µg RNA was used for cDNA synthesis with RealMasterMix (Eppendorf, Hamburg, Germany). PCR amplification was carried out in a total volume of 10 µL, containing 1 µL cDNA solution, 4.5 µL SYBR solution Mastermix (Eppendorf, Hamburg, Germany), 0.5 µL each primer (Primerdesign, Southampton, United Kingdom), 4 µL of nuclease-free water. β-actin was quantified, and used for the normalization of expression values of the other genes. Fluorescence signals measured during the amplification were considered positive if the fluorescence intensity was more than 20-fold greater than the standard deviation of the baseline fluorescence. The ΔΔ*C*T method of relative quantification was used to determine the fold change in expression. Here, the threshold cycle (*C*T) values of the target mRNAs were first normalized to the *C*T values of the internal control, β-actin, in the same samples (Δ*C*T = *C*Ttarget−*C*Tcon), and then further normalized with the internal control (T0). Assays were run in triplicate with Universal PCR Master Mix on MasterCycler realplex (Eppendorf, Hamburg, Germany) PCR system. The sequence of primer pairs were as follows: β-Actin (NM_000600), Nox 4 (NM_016931), Urat1 (SLC22A12) (NM_144585.3). The sequences are reported in Table 1.

**Table 1:** Sequences of gene-specific primers used in qRT-PCR.

Primers	Forward	Reverse
NADPH oxidase 4 (NOX 4)	cacagacttggctttggatttc	ggatgacttatgaccgaaatgatg
SLC22A12 (URAT 1)	gcccgacaccatccaaga	ttcctctgaccgtcccatc
β-Actin	catcccccaaagttcacaat	agtggggtggcttttaggat

### Statistical analysis

Statistical analysis was performed with the InStat software package version 2.01 (Graph Pad, San Diego, CA, USA). The one-way analysis of variance (ANOVA) and the Tukey-Kramer multiple comparison test were used to test the significance of differences. Results are expressed as mean±SEM and are the expression of at least three experiments, with two wells for each experiment. Differences were considered statistically significant if p<0.05.

## Results

### Cell viability

**Figure 1:**
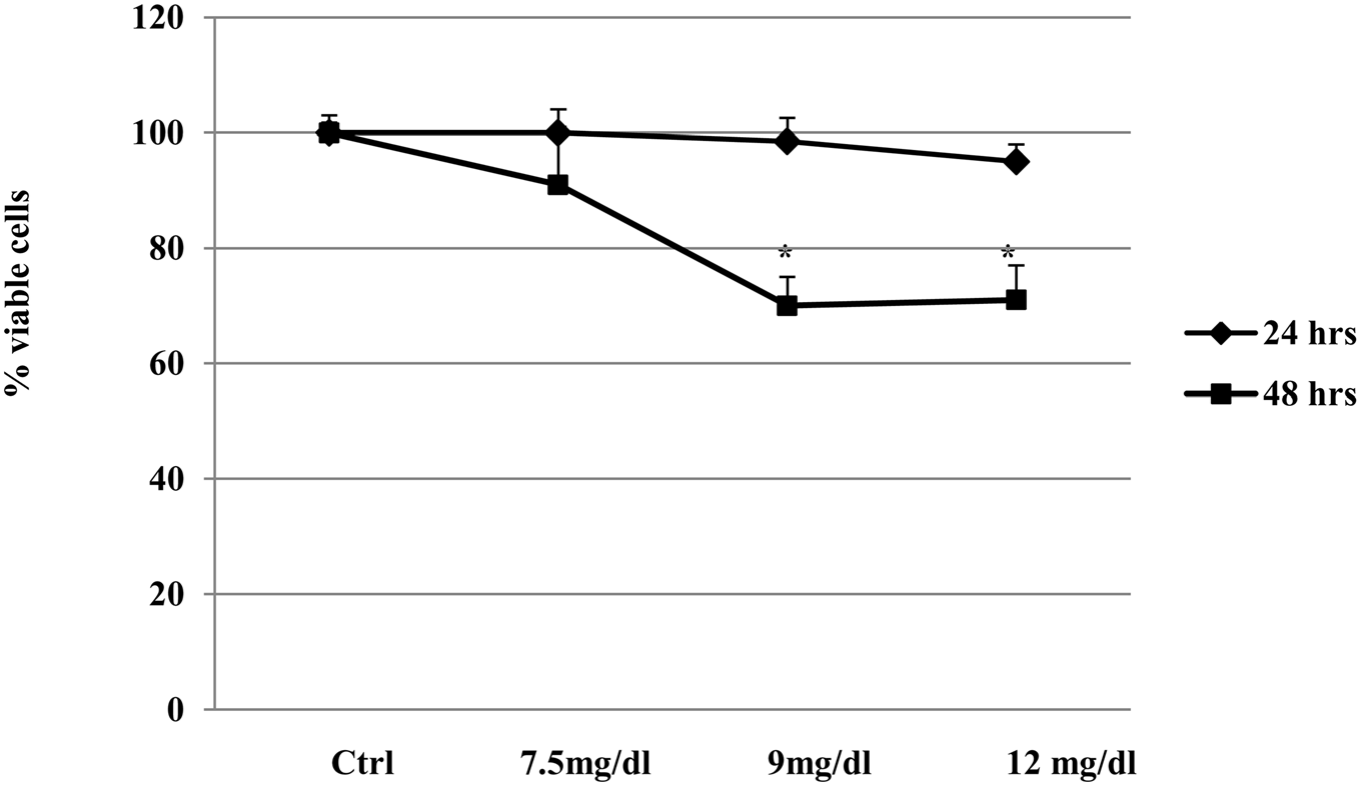
UA decreases cell viability in proximal tubular epithelial cells. Effects of increasing doses of UA on HK-2 viability at 24 and 48 hours (MTT test). A decrease in cell viability was observed with higher concentrations (9–12 mg/dl). For each treatment group the number of cells at t = 0 served as baseline value 100% and was used to express the percentage of living cells. Data shown as mean ± SEM; *p<0.05 vs. Ctrl. UA, uric acid; Ctrl, control untreated cells.

To examine the apoptotic effects of UA, cells were treated with different UA concentrations (7.5 to 12 mg/dl) for 24–48 hours. During the course of the experiments, the majority of cells remained viable and attached to culture dishes in control cultures over the 48-hour period. When UA was used at 7.5 mg/dl, 91±10% of cells were viable at 48 hours (p<0.05 vs. Ctrl). With 9–12 mg/dl UA, cell viability was unchanged at 24 hours but significantly decreased at 48 hours (70±5% of HK-2 cells viable, p<0.01 vs. Ctrl) (Fig. 1).

### Effects of UA on apoptosis of renal tubular cells

**Figure 2:**
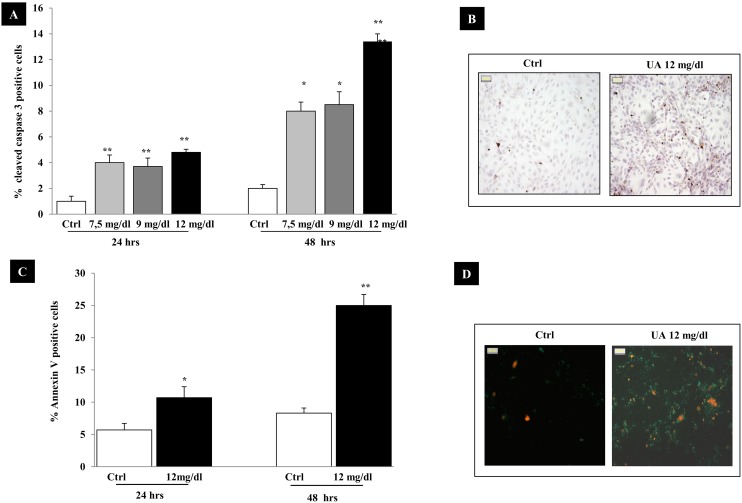
UA triggers apoptosis in proximal tubular epithelial cells. HK-2 were exposed for 24–48 hours to UA (7.5–12 mg/dl). Apoptosis was evaluated by anti-cleaved caspase 3 (A) antibody and by annexin V/propidium iodide (C) and examined under microscope. Apoptotic cells were expressed as percent of total cells counted (>400 cells for each condition). Photos are representative of cleaved caspase 3 immunostaining (B) and of Annexin V/propidium iodide staining (D) (magnification x400). Data shown as mean ± SEM; *p<0.05, **p<0.001, vs. Ctrl. UA, uric acid; Ctrl, control untreated cells.

To determine whether UA promotes apoptosis in HK-2 cells, cells were incubated for 24–48 hours with different concentrations of UA in multiple experiments and subjected to apoptosis assay. Experiments were performed under identical conditions (Fig. 2). During the course of these experiments only 1±0.4% of the untreated cells were cleaved caspase 3 positive and this percentage increased slightly at 48 hours (+1±0.3%) (Fig. 2A). UA induced a dose and time-dependent rise in apoptotic index with a 4-fold increase observed at 7.5–9 mg/dl of UA (p<0.001 vs. Ctrl) and a 7-fold increase at 12 mg/dl (p<0.0001 vs. Ctrl) after 48 hours. The proapoptotic effect of 12 mg/dl UA was shown also by the use of annexin V/propidium iodide staininig. By this technique, the percentage of apoptotic cells increased by ∼2-folds at 24 hours (UA 10.7±1.7% vs. 5.7±1% Ctrl, p<0.05) and ∼3 folds at 48 hours (UA 25±1.7%, Ctrl 8.3±0.8%, p<0.001) as compared to untreated cells (Fig. 2B).

### Effects of caspase inhibition

**Figure 3:**
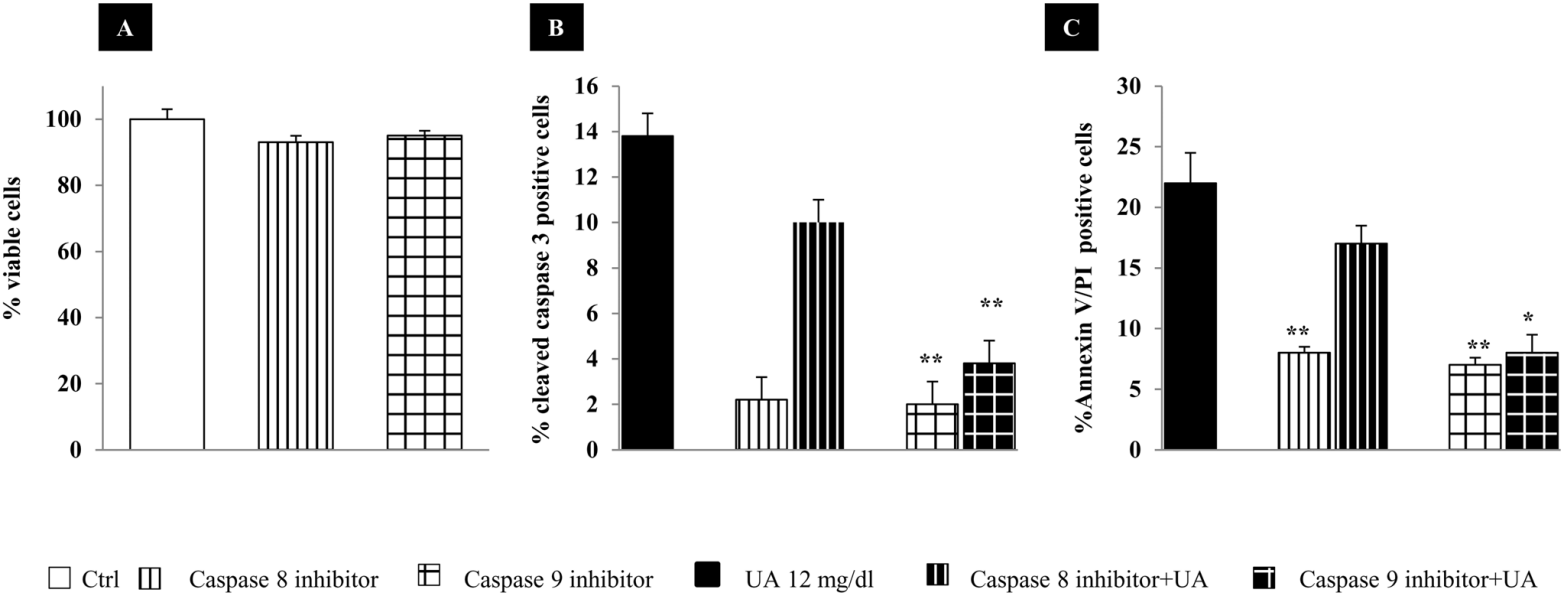
Effects of caspase inhibitor 8 (Z-IEDT-FMK) and caspase inhibitor 9 (Z-LEHD-FMK) (50 µM) on cell viability and 12 mg/dl UA-induced apoptosis, at 48 hours. No significant effect of Caspases inhibitors on cell viability was observed by MTT (A). Apoptosis was evaluated by anti-cleaved caspase 3 antibody (B) and by annexin V/propidium iodide (C) and examined under microscope. Positive cells were expressed as percentage of total cells counted (∼350 cells for each condition). Data shown as mean ± SEM of three different experiments. *p<0.01, **p<0.0001 vs. Ctrl. UA, uric acid; Ctrl, control untreated cells.

Two main pathways leading to apoptosis through caspase activation have been described. On one way, the “extrinsic pathway” is activated by receptors of the TNFR superfamily through mechanisms that involve the adapter protein FADD, which activates the initiator caspase-8. On the other way, the “intrinsic pathway” is activated by mitochondrial proteins through the action of the initiator caspase-9. Since the inhibition of caspase functions has been shown to block the development of programmed cell death [29], we evaluated whether UA-induced HK-2 cell apoptosis could be prevented by treatment with different inhibitors. First, we ruled out any toxic effects of 50 µM caspase-8, -9 inhibitors (Fig. 3A). The antiapoptotic effects were not the same for all inhibitors (Fig. 3B, C). Changes in cellular morphology induced by UA at 48 hours were no longer observed after the addition to the culture medium of caspase-9 (Z-LEHD-FMK) (p<0.0001 vs. Ctrl), but not caspase-8 (Z-IEDT-FMK) inhibitor. These results suggest a main involvement of the intrinsic mitochondrial pathway in UA induced apoptosis.

### Effect of UA on the disruption of the mitochondrial transmembrane potential and apoptosis proteins

**Figure 4:**
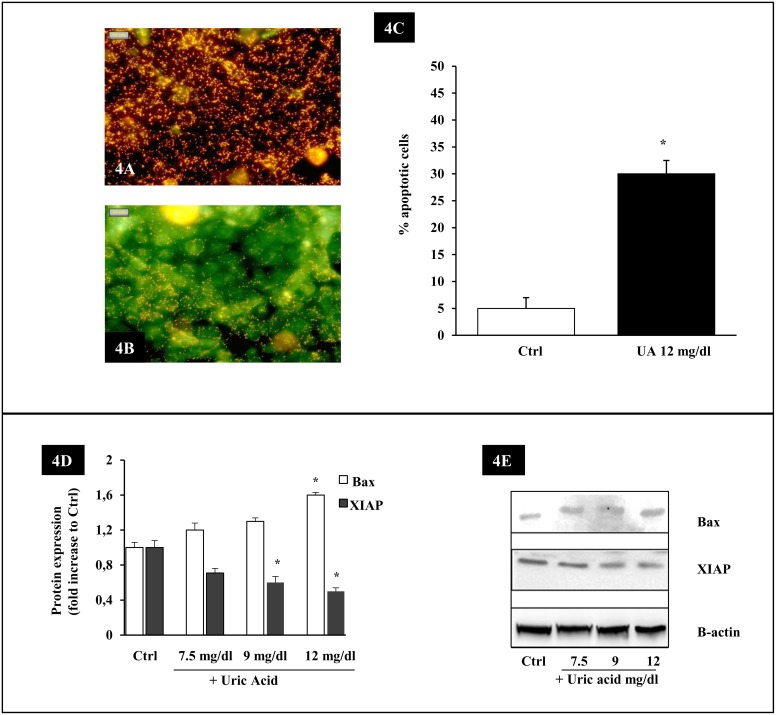
Effect of UA on the disruption of the mitochondrial transmembrane potential and apoptosis proteins. Upper panel: UA causes an alteration in the mitochondrial membrane potential, detected by the MitoCapture technique. (A) In control cells, the cathionic dye accumulates in the mitochondria and it fluoresces red. (B) In UA treated cells the mitochondrial membrane potential is dissipated and the Mitocapture dye is dispersed into the cell as green fluorescent monomers. (C) UA induces a significant increase in green cells (magnification x 400). Data shown as mean ± SEM, *p<0.05. Lower panel: Effect of UA on proteins implicated in apoptosis. HK-2 cells were exposed for 48 hours to 7.5–12 mg/dl UA and cell lysates were separated by SDS-PAGE and immunoblotted with antibodies to Bax and XIAP. (D) The graph shows Bax and XIAP changes over Ctrl. (E) Representative immunoblots from 3 different experiments are shown. Data shown as mean ± SEM, *p<0.05. Ctrl, Control untreated cells; UA, uric acid.

The intrinsic apoptotic pathway hinges on the balance of activity between pro- and anti-apoptotic members of the Bcl-2 superfamily of proteins which act to regulate the integrity of the mitochondrial membrane. To study the role of the apoptotic intrinsic pathway, we examined the mitochondrial membrane integrity by MitoCapture (Fig. 4A–C) and the expression of Bax and X-linked inhibitor of apoptosis (XIAP), an endogenous inhibitor of caspases (Fig. 4D, E).

We observed that 30±2.5% of UA treated cells presented an altered mitochondrial transmembrane potential (p<0.05 vs. Ctrl) (Fig. 4A–C). Densitometry of immunoblots from separate experiments showed that UA induced a significant dose dependent increase in the expression of Bax (+60% with respect to Ctrl; p<0.05) (Fig. 4D, E). In addition, the expression of XIAP was decreased in whole-cell lysates from HK-2 cells incubated with 7.5–12 mg/dl UA at 48 hours when compared with Ctrl (Fig. 4D, E).

### Effect of UA on the production of reactive oxygen species (ROS)

**Figure 5:**
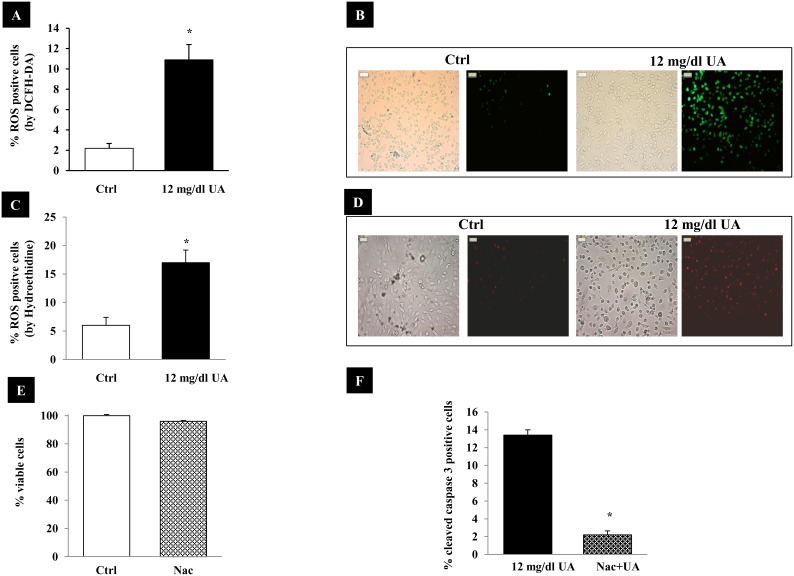
ROS production in UA treated cells. (A, B) The panels show representative images of DCFH-DA and HE accumulation in cells after UA treatment. Pictures show the same fields in bright and fluorescence light (magnification x 400). (C, D) The graphics show a quantitative analysis of ROS production. The results are reported as percentage of DCFH-DA and HE positive cells. For each condition ∼350 cells were counted; (D) 10 mMol NAC did not alter cell viability, assessed by MTT test (E) NAC decrease apoptosis of HK-2 cells treated with 12 mg/dl UA. Cells were treated with NAC (10 mM) and UA for 48 hours. Cells were stained by anti-cleaved caspase 3 antibody and examined under microscope. Apoptotic cells were expressed as percent of total cells counted (∼350 cells). Data shown as mean ± SEM, * = p<0.001 vs. Ctrl. DCFH-DA, 2′–7′ dichlorofluorescein-diacetate, HE Hydroethidine; Ctrl, control untreated cells; UA, uric acid; NAC = N-acetyl-cysteine.

To investigate the possible mechanisms underlying the effects of UA on Hu-PTCs, we measured the intracellular ROS levels in HK-2 cells stimulated with 12 mg/dl UA. Intracellular ROS levels were investigated by DCFH-DA (Fig. 5A, B) and Hydroethidine (Fig. 5C–D). ROS generation was ∼3–5 fold increased after 24 hours from the stimulation with UA (Fig. 5A–D). Oxidative stress promotes cell death and ROS are involved in proliferation and apoptosis. To verify the proapoptotic effects of ROS, cells were preincubated with a non cytotoxic dose of N-acetyl-cysteine (NAC) (10 mM), as confirmed by MTT test (p = NS vs. Ctrl) (Fig. 5E). NAC is a ROS scavenger and significantly blunted UA apoptosis (Fig. 5F). These data indicate that UA can immediately induce intracellular ROS production and that they are involved in UA apoptosis.

### Effects of UA on gene expression of NAD(P)H oxidase

**Figure 6:**
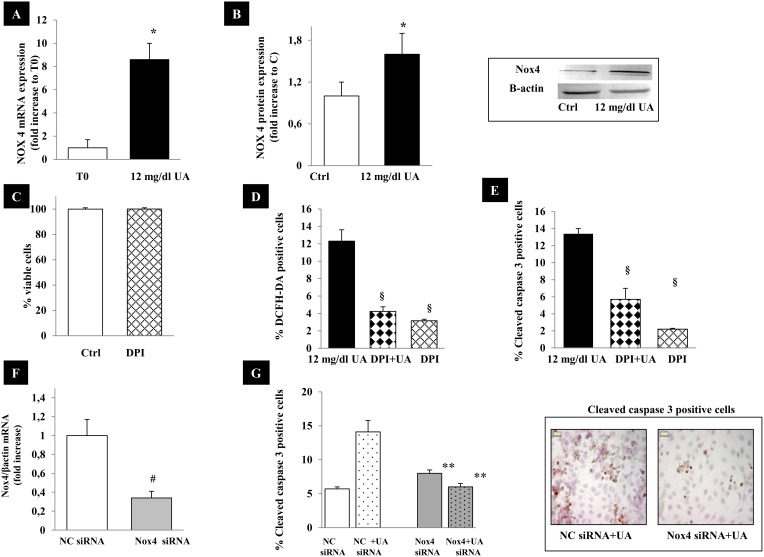
Effects of UA on NOX 4 expression. (A) NOX 4 expression was evaluated by real time-PCR at time 0 and 5 hours after 12 mg/dl UA incubation. The results are reported as fold increase to T0; (B) NOX 4 expression was evaluated by western blot at 24 hours. The results are reported as fold increase to Ctrl. (C) 10 10 µM DPI had no cytotoxic effects on HK-2 as assessed by MTT test. (D) DPI inhibits ROS production in UA treated cells. The results are reported as percentage of DCFH-DA positive cells. For each condition ∼350 cells were counted; (E) Effects of NOX 4 inhibition on apoptosis. HK-2 cells were treated with DPI (10 µM) and 12 mg/dl UA. Cells were immunostained by anti-cleaved caspase 3 antibody and examined under microscope. For each condition ∼350 cells were counted. Apoptotic cells were expressed as percent of total cells counted. (F) HK-2 were transfected with 60 nM nonspecific negative control siRNA (NC siRNA) or Nox 4 specific siRNA. Gene expression was evaluated by real time PCR after 24 hours. (G) Down-regulation of Nox 4 by RNA interference decreased UA induced apoptosis in Nox4 siRNA respect to NC siRNA. Pictures show the effects of Nox 4 silencing on cleaved caspase 3 expression (magnification x 1000). Data shown as mean ± SEM. *p<0.05 vs. time 0, § = p<0.005 vs. cells treated with 12 mg/dl UA, **p<0.001 vs. NC siRNA+UA, #p<0.01 vs. NC**.** DPI, diphenylene iodonium; UA, uric acid; T0, time 0; Ctrl, control untreated cells. NC, Negative Control.

To further investigate the mechanisms underlying the induction of ROS release, we examined the effects of UA on the expression of NOX 4, a member of the NAD(P)H oxidase complex, which generates ROS and plays a critical role in mediating mitochondrial dysfunction and apoptosis in renal tubular cells. RNA from HK-2 cells incubated for 5 hours with 12 mg/dl UA, was subjected to real time-PCR and western blot. As shown in Fig. 6A and B, UA induced an increase in NOX 4 mRNA (by ∼8.6 folds, p<0.05) and protein (by ∼1.6 folds, p<0.05). When HK-2 were pretreated with diphenylene iodonium (DPI), a NADPH oxidase inhibitor, ROS production (Fig. 6D,.–70% vs. UA treated cells, p<0.005) as well as UA induced apoptosis (Fig. 6E, –50% vs. UA treated cells, p<0.005) were significantly blunted. DPI had no effects on cell viability (Fig. 6C). The involvement of Nox4 in UA induced apoptosis was supported by its silencing by RNA interference. When Nox 4 knockdown cells were exposed to UA, apoptosis was significantly decreased (∼50%, p<0.0001) with respect to cells transfected with nonspecific negative control siRNA (Fig. 6G).

Collectively, these data support the involvement of NOX4 oxidase in ROS generation and their role in UA induced apoptosis.

### Involvement of MAPKs in the UA-induced apoptosis

**Figure 7:**
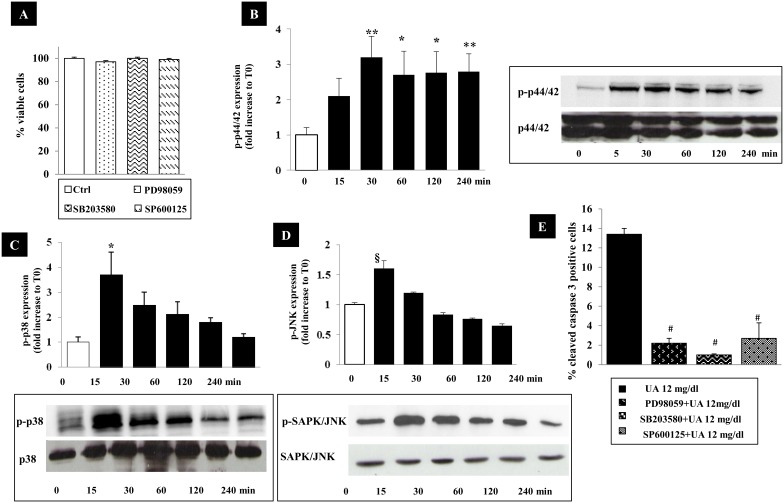
Time course of mitogen-activated protein kinase (MAPK) activity by UA. (A) Effects of MAPKs inhibitors on HK-2 viability evaluated by MTT. HK-2 were treated for different time intervals (0–240 minutes) with 12 mg/dl UA. Then, phosphorylated p44/42 (B), p38 (C), SAPK/JNK (D) MAPKs were detected by Western blot. The pictures shown are representative of 3 experiments. The graphs represent relative phospho-MAPKs protein abundance normalized to MAPKs and data are expressed as fold increase respect to basal value (T0) and as means ± SEM of 3 independent experiments. (E) Effects of MAPKs inhibitors on UA-induced apoptosis. HK2 were treated with PD 98059 (a p44/42 MAPK inhibitor), SB 203580 (a p38 MAPK inhibitor), SP 600125 (a SAPK/JNK inhibitor), for 60 min before treatment with 12 mg/dl UA. Values are means ± SEM of 3 independent experiments with duplicate wells. *p<0.05, **p<0.01, §p<0.0001 vs. Time 0. #p<0.0001 vs. UA treated cells. UA, uric acid; min, minutes; T0, time 0.

Mitogen-activated protein kinases (MAPKs) is an essential intracellular signalling pathway involved in growth and differentiation, development, apoptosis and a series of physiological and pathological processes. MAPK family includes three primary members: p44/42 MAPK (ERK), p38 MAPK and SAPK/JNK. We evaluated the effects of 12 mg/dl UA on MAPKs phosphorylation and their involvement in apoptosis. Fig. 7(A–C) shows the effect of UA on the activation of p44/42 MAPK (pERK), p38 MAPK and SAPK/JNK, over a 4-hours incubation period. In UA treated cultures, the level of phosphorylation of p38 MAPK and SAPK/JNK increased ∼1.6–3.7 folds (p<0.05–0.001 vs. T0) after 15 min incubation period and then returned to the control level. Contrariwise, p44/42 MAPK phosphorylation increased by 2-folds after 15 minutes and it was unchanged over the time course (p<0.05–0.01 vs. T0).

As a next step, we examined the effects of the MAPK inhibitors PD 98059 (p44/42 MAPK) SB 203580 (p38 MAPK) and SP 600125 (SAPK/JNK) on UA-induced apoptosis. The concentrations used had no effects on cell viability (Fig. 7D). Fig. 7E shows that all the MAPK inhibitors prevented apoptosis (# p<0.0001 vs. UA treated cells).

### Effects of inhibition urate transporters on UA-induced apoptosis

**Figure 8:**
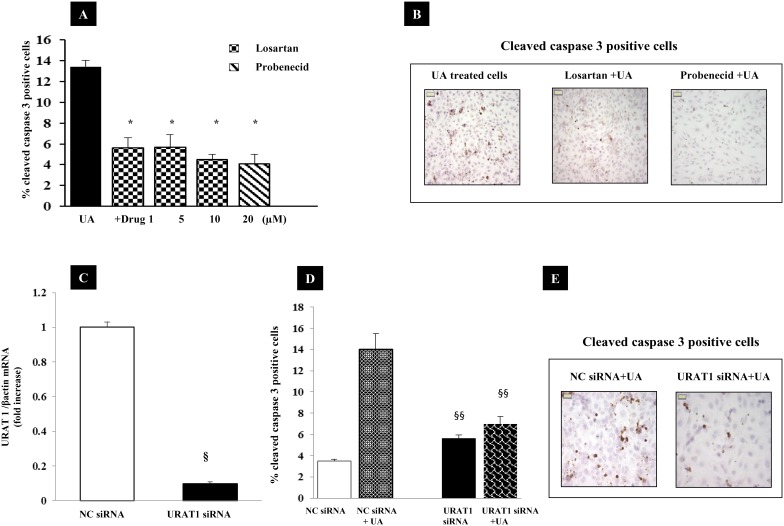
Efficacy of URAT1 inhibition. (A) Effects of Losartan and Probenecid on UA-induced apoptosis. HK-2 were treated with 1–10 µM Losartan or 20 µM Probenecid and 12 mg/dl UA for 48 hours. Cells were stained by anti-cleaved caspase 3 antibody and examined under microscope. Apoptotic cells were expressed as percent of total cells counted (∼350 cells). Data shown as mean ± SEM, * = p<0.0001 vs. Cells incubated with 12 mg/dl UA. (B) Pictures show the effects of different treatments on cleaved caspase 3 expression (magnification x 400). (C) HK-2 were transfected with 60 nM nonspecific negative control siRNA (NC siRNA) or URAT 1 specific siRNA. Gene expression was evaluated by real time PCR after 24 hours. (D) Down-regulation of URAT 1 by RNA interference decreased UA induced apoptosis in URAT1 siRNA respect to NC siRNA. (E) Pictures show the effects of URAT1 silencing on cleaved caspase 3 expression (magnification x 1000). Data shown as mean ± SEM. §p<0.001 vs. NC siRNA, §§p<0.0001 vs NC siRNA exposed to UA. Abbreviations: UA, Uric acid; NC, Negative Control.

To determine whether UA-induced apoptosis was affected by UA internalization we studied the effects of Probenecid (20 µM), an inhibitor of urate transport via the organic anion transporter (OAT) family [30] and Losartan (1–10 µM) a specific inhibitor of URAT1 (URAT1:*SLC22A12*) on UA-induced apoptosis [31]. Both Probenecid and Losartan blunted apoptosis by ∼60–70% (p<0.0001 vs. UA-treated cells) (Fig. 8A, B).

### Effects of siRNA URAT 1 transfection on UA-induced apoptosis

To confirm the direct involvement of URAT 1 in UA apoptosis, its expression was down-regulated after 24 hours of siRNA transfection (Fig. 7C). When URAT 1 knockdown HK-2 cells were exposed to UA, apoptosis was significantly decreased (∼50%, p<0.0001) with respect to cells transfected with nonspecific negative control siRNA (Fig. 8D, E).

## Discussion

The present study shows that UA promotes a dose-dependent apoptotic damage in Hu-PTCs as evaluated by multiple apoptosis-associated determinations. Particularly, these effects are evident at UA levels that are commonly observed during moderate hyperuricemia in humans and are blunted by several methods, including knock out for URAT 1 and pharmacological interventions to block URAT 1.

Oxidative stress in human tubular cells has emerged as a major cause of renal damage in different pathophysiological conditions, such as ischemia-reperfusion, chronic renal failure, hypertension, and diabetic nephropathy [32]. In this study we show that apoptotic events that occur when Hu-PTCs are cultivated under high UA levels are dependent on ROS generation and up-regulation of NOX 4 NADPH oxidase. In the kidney, ROS are primarily produced by NADPH oxidases [33]–[35]. All components of the NADPH oxidase complex, including p22phox, p47phox, and p67phox as well as the NOX isoforms 1, 2, and 4, are expressed in tubular cells [36]. Even if the function of NADPH oxidase in regulating physiological and pathophysiological processes in the kidney remains largely unknown, there is a wide array of evidence that NOX-derived ROS modulate renal epithelial ion transport [36], extracellular matrix protein synthesis [37]–[38] and apoptosis [39]–[42]. The UA promoted apoptotic damage shown here might interact with the apoptotic pathway already activated in several chronic kidney diseases and favour disease progression. Since the redox-dependent effects of UA in tubular cells were not mediated by the redox chemistry of the urate compound but by the activation of intracellular oxidant production, our results offer also a possible explanation for the paradox by which urate, as a chemical antioxidant [11], drives oxidative stress.

Inhibition of NADPH-oxidase is protective in several experimental models of acute and chronic kidney disease [37]–[38]. Our findings strongly offer an explanation for the renal protective effects of xantino-oxidase inhibitors [43]. In our study, we observed that UA-induced apoptosis was prevented both by NADPH oxidase inhibition and the antioxidant NAC suggesting a major role of UA in inducing oxidative stress in the tubulointerstitium. The shown effects of UA on Hu-PTCs could be critically addictive to mechanisms of cell loss which have been already described to be activated in chronic renal diseases, hypertension, and diabetes [44]–[45].

Concurrent with the increased ROS formation, we observed UA-induced alterations in mitochondrial membrane potential and in Bax expression. Upon induction of apoptosis, Bax interacts with the mitochondrial membrane and causes the opening of the mitochondrial voltage-dependent anion channel. This provokes the release of cytochrome c and other pro-apoptotic factors from the mitochondria, leading to activation of caspase 9 [46]. In our study, UA apoptosis was blunted by caspase 9 inhibition, suggesting a predominant role of the intrinsic pathway in UA-induced apoptosis. Besides, we observed that the expression of XIAP, an endogenous inhibitor of caspases, was decreased by high UA medium. To our knowledge, this is the first demonstration of XIAP regulation by UA. XIAP inhibits caspases primarily by preventing cleavage of pro-caspases but may also directly inhibit activated caspases [47]. A mechanism by which UA might cause cell death by downregulating endogenous inhibitors of caspases is an attractive one and needs to be further explored.

In Hu-PTCs, extracellular-signal regulated kinases 1 and 2 (ERK1/2), c-jun NH2-terminal kinase (JNK1/2) and p38 are expressed and are able to influence cell death. However, little is known about the effect of UA on MAPKs activation. Han et al. showed that p38 and JNK phosphorylation induced by elevated UA were responsible of inhibition of rabbit prossimal tubular cells proliferation [48] and Quan H. et al observed that when HK-2 were stimulated with 600 µM UA for 48 h, ERK1/2, several proapoptotic proteins and MAPKs were up-regulated [22]. In our study, we present evidence for the first time, that UA induced MAPKs activation and apoptosis were interrelated and that this event was blunted by MAPKs inhibitors. Taken together, these data corroborate the hypothesis that UA has a pathogenetic role in the development of renal damage.

In previous studies the inhibition of transmembrane transport of urate by probenecid and benzbromarone (two structurally unrelated OAT inhibitors of the transmembrane transport of urate) prevented ROS-induced generation in adipocytes suggesting that UA must enter the cell to induce ROS production [11]. In the present study, UA induced apoptosis was prevented by addition of aspecific and selective URAT1 blockers such as probenecid and losartan. In addition, knockdown of URAT-1 by means of specific siRNA demonstrated that activation of mitochondrial pathway of apoptosis was URAT-1 dependent in HK-2 cells. Thus our data strongly suggest that UA internalization in HK-2 is necessary to exert cell damage and apoptotic loss.

Clinical and experimental data seem to support a role for UA lowering treatment in providing renal protection [49]. Experimental studies on rat models of mild hyperuricemia have suggested that pharmacologic reduction of UA levels by means of different drugs, such as allopurinol, benzodiarone or febuxostat can prevent the development of systemic hypertension, renal arteriolopathy and glomerular hypertension [18], [20], [50] but again, biological mechanism(s) underlying this effects are still incompletely understood. While these studies have occasionally been taken to indicate that UA lowering drugs such as allopurinol and febuxostat exert their renal protective effect through a specific inhibition of XOR-induced oxidants rather than through their direct action on UA levels, our data better may help clarify the role of internalization of UA as a trigger of a cascade leading to a caspase-dependent apoptosis.

URAT-1 pharmacologic blockade is thought to be responsible for the observed uricosuric action of Losartan [31]. Results from the LIFE and RENAAL studies [51]–[53], suggest that favourable cardiorenal effects observed with Losartan may be due, in a large part, to its effect on UA. These findings integrated with results presented here, suggest that the protection against pro-oxidant agents might be obtained by the reduction or neutralization of the agent itself.

In conclusion, our observations suggest that URAT-1 inhibition might modulate the development of renal tubular damage thorough the blockade of the redox-dependent stress triggered by UA exposition. These results may be of relevance to define more effective therapeutic strategies for cardiovascular and renal protection in hyperuricemic states.

## References

[1] Obermayr RP, Temml C, Gutjahr G, Knechtelsdorfer M, Oberbauer R, et al (2008) Elevated uric acid increases the risk for kidney disease. J Am Soc Nephrol 19:2407–2013. 18799720 10.1681/ASN.2008010080 PMC2588108

[2] Weiner DE, Tighiouart H, Elsayed EF, Griffith JL, Salem DN, et al (2008) Uric acid and incident kidney disease in the community. J Am Soc Nephrol 19:1204–1211. 18337481 10.1681/ASN.2007101075 PMC2396939

[3] Iseki K, Oshiro S, Tozawa M, Iseki C, Ikemiya Y, et al (2001) Significance of hyperuricemia on the early detection of renal failure in a cohort of screened subjects. Hypertens Res 24:691–697. 11768729 10.1291/hypres.24.691

[4] Johnson RJ, Nakagawa T, Jalal D, Sánchez-Lozada LG, Kang DH, et al (2013) Uric acid and chronic kidney disease: which is chasing which? Nephrol Dial Transplant 28:2221–2228. 23543594 10.1093/ndt/gft029 PMC4318947

[5] Viazzi F, Leoncini G, Ratto E, Falqui V, Parodi A, et al (2007) Mild hyperuricemia and subclinical renal damage in untreated primary hypertension. Am J Hypertens 20:1276–1282. 18047917 10.1016/j.amjhyper.2007.08.010

[6] Jalal DI, Rivard CJ, Johnson RJ, Maahs DM, McFann K, et al (2010) Serum uric acid levels predict the development of albuminuria over 6 years in patients with type 1 diabetes: findings from the Coronary Artery Calcification in Type 1 Diabetes study. Nephrol Dial Transplant 25:1865–1869. 20064950 10.1093/ndt/gfp740 PMC2902891

[7] Zoppini G, Targher G, Chonchol M, Ortalda V, Abaterusso C, et al (2012) Serum uric acid levels and incident chronic kidney disease in patients with type 2 diabetes and preserved kidney function. Diabetes Care 35:99–104. 22028277 10.2337/dc11-1346 PMC3241303

[8] Ohno I, Hosoya T, Gomi H, Ichida K, Okabe H, et al (2001) Serum uric acid and renal prognosis in patients with IgA nephropathy. Nephron 87:333–339. 11287777 10.1159/000045939

[9] Syrjänen J, Mustonen J, Pasternack A (2001) Hypertriglyceridaemia and hyperuricaemia are risk factors for progression of IgA nephropathy. Nephrol Dial Transplant 15:34–42. 10.1093/ndt/15.1.34 10607765

[10] Sánchez-Lozada LG, Lanaspa MA, Cristóbal-García M, García-Arroyo F, Soto V, et al (2012) Uric acid-induced endothelial dysfunction is associated with mitochondrial alterations and decreased intracellular ATP concentrations. Nephron Exp Nephrol 121:e71–78. 23235493 10.1159/000345509 PMC3656428

[11] Sautin YY, Nakagawa T, Zharikov S, Johnson RJ (2007) Adverse effects of the classic antioxidant uric acid in adipocytes: NADPH oxidase-mediated oxidative/nitrosative stress. Am J Physiol Cell Physiol 293:C584–C596. 17428837 10.1152/ajpcell.00600.2006

[12] Ray PD, Huang BW, Tsuji Y (2012) Reactive oxygen species (ROS) homeostasis and redox regulation in cellular signaling. Cell Signal 24:981–990. 22286106 10.1016/j.cellsig.2012.01.008 PMC3454471

[13] Sautin YY, Johnson RJ (2008) Uric acid: the oxidant-antioxidant paradox. Nucleosides Nucleotides Nucleic Acids 27:608–661. 18600514 10.1080/15257770802138558 PMC2895915

[14] Nieto FJ, Iribarren C, Gross MD, Comstock GW, Culter RG (2000) Uric acid and serum antioxidant capacity: a reaction to atherosclerosis? Atherosclerosis 148:131–139. 10580179 10.1016/s0021-9150(99)00214-2

[15] Kanellis J, Kang DH (2005) Uric acid as a mediator of endothelial dysfunction, inflammation, and vascular disease. Semin Nephrol 25:39–42. 15660333 10.1016/j.semnephrol.2004.09.007

[16] Kanellis J, Watanabe S, Li JH, Kang DH, Li P, et al (2003) Uric acid stimulates monocyte chemoattractant protein-1 production in vascular smooth muscle cells via mitogen-activated protein kinase and cyclooxygenase-2. Hypertension 41:1287–1293. 12743010 10.1161/01.HYP.0000072820.07472.3B

[17] Kang DH, Han L, Ouyang X, Kahn AM, Kanellis J, et al (2005) Uric acid causes vascular smooth muscle cell proliferation by entering cells via a functional urate transporter. Am J Nephrol 25:425–433. 16113518 10.1159/000087713

[18] Sánchez-Lozada LG, Tapia E, Santamaría J, Avila-Casado C, Soto V, et al (2005) Mild hyperuricemia induces vasoconstriction and maintains glomerular hypertension in normal and remnant kidney rats. Kidney Int 67:237–247. 15610247 10.1111/j.1523-1755.2005.00074.x

[19] Kang DH, Park SK, Lee IK, Johnson RJ (2005) Uric acid-induced C-reactive protein expression: implication on cell proliferation and nitric oxide production of human vascular cells. J Am Soc Nephrol 16:3553–3562. 16251237 10.1681/ASN.2005050572

[20] Feig DI, Mazzali M, Kang DH, Nakagawa T, Price K, et al (2006) Serum uric acid: a risk factor and a target for treatment? J Am Soc Nephrol 17 Suppl 2: 4):S69–73. 16565251 10.1681/ASN.2005121331

[21] Cirillo P, Gersch MS, Mu W, Scherer PM, Kim KM, et al (2009) Ketohexokinase-dependent metabolism of fructose induces proinflammatory mediators in proximal tubular cells. J Am Soc Nephrol 20:545–553. 19158351 10.1681/ASN.2008060576 PMC2653686

[22] Quan H, Peng X, Liu S, Bo F, Yang L, et al (2011) Differentially expressed protein profile of renal tubule cell stimulated by elevated uric acid using SILAC coupled to LC-MS. Cell Physiol Biochem 27:91–98. 21325826 10.1159/000325209

[23] Gasse P, Riteau N, Charron S, Girre S, Fick L, et al (2009) Uric acid is a danger signal activating NALP3 inflammasome in lung injury inflammation and fibrosis. Am J Respir Crit Care Med 179:903–913. 19218193 10.1164/rccm.200808-1274OC

[24] Wang C, Pan Y, Zhang QY, Wang FM, Kong LD (2012) Quercetin and allopurinol ameliorate kidney injury in STZ-treated rats with regulation of renal NLRP3 inflammasome activation and lipid accumulation. PLoS One 7:e38285. 22701621 10.1371/journal.pone.0038285 PMC3372527

[25] Havasi A, Borkan SC (2011) Apoptosis and acute kidney injury. Kidney Int 80:29–40. 21562469 10.1038/ki.2011.120 PMC4625984

[26] Ortiz A (2000) Nephrology forum: apoptotic regulatory proteins in renal injury. Kidney Int 58:467–485. 10886604 10.1046/j.1523-1755.2000.00188.x

[27] Lhotta K, Gruber J, Sgonc R, Fend F, König P (1998) Apoptosis of tubular epithelial cells in familial juvenile gouty nephropathy. Nephron 79:340–344. 9678437 10.1159/000045060

[28] Capellino S, Montagna P, Villaggio B, Soldano S, Straub RH, et al (2008) Hydroxylated estrogen metabolites influence the proliferation of cultured human monocytes: possible role in synovial tissue hyperplasia. Clin Exp Rheumatol 26:903–909. 19032826

[29] Nicholson DW (1996) ICE/CED-3-like proteases as therapeutic targets for the control of inappropriate apoptosis. Nat Biotech 14:297–301. 10.1038/nbt0396-297 9630889

[30] Miyazaki H, Sekine T, Endou H (2004) The multispecific organic anion transporter family: properties and pharmacological significance. Trends Pharmacol Sci 25:654–662. 15530644 10.1016/j.tips.2004.10.006

[31] Hamada T, Ichida K, Hosoyamada M, Mizuta E, Yanagihara K, et al (2008) Uricosuric action of losartan via the inhibition of urate transporter 1 (URAT 1) in hypertensive patients. Am J Hypertens 21:1157–1162. 18670416 10.1038/ajh.2008.245

[32] Palm F, Nordquist L. (2011) Renal tubulointerstitial hypoxia: cause and consequence of kidney dysfunction. Clin Exp Pharmacol Physiol 38:474–480. 21545630 10.1111/j.1440-1681.2011.05532.x PMC3124566

[33] Sedeek M, Callera G, Montezano A, Gutsol A, Heitz F, et al (2010) Critical role of Nox4-based NADPH oxidase in glucose-induced oxidative stress in the kidney: implications in type 2 diabetic nephropathy. Am J Physiol Renal Physiol 299:F1348–1358. 20630933 10.1152/ajprenal.00028.2010

[34] Gorin Y, Block K, Hernandez J, Bhandari B, Wagner B, et al (2005) Nox4 NAD(P)H oxidase mediates hypertrophy and fibronectin expression in the diabetic kidney. J Biol Chem 280:39616–39626. 16135519 10.1074/jbc.M502412200

[35] Gill PS, Wilcox CS (2006) NADPH oxidases in the kidney. Antioxid Redox Signal 8:1597–1607. 16987014 10.1089/ars.2006.8.1597

[36] Schreck C, O'Connor PM (2011) NAD(P)H oxidase and renal epithelial ion transport. Am J Physiol Regul Integr Comp Physiol 300:R1023–1029. 21270341 10.1152/ajpregu.00618.2010 PMC3094034

[37] Barnes JL, Gorin Y (2011) Myofibroblast differentiation during fibrosis: role of NAD(P)H oxidases. Kidney Int 79:944–956. 21307839 10.1038/ki.2010.516 PMC3675765

[38] New DD, Block K, Bhandhari B, Gorin Y, Abboud HE (2011) IGF-I increases the expression of fibronectin by Nox4-dependent Akt phosphorylation in renal tubular epithelial cells. Am J Physiol Cell Physiol 302: C122–130, 2012. 10.1152/ajpcell.00141.2011 PMC3328902 21940672

[39] Susztak K, Raff AC, Schiffer M, Böttinger EP (2006) Glucose-induced reactive oxygen species cause apoptosis of podocytes and podocyte depletion at the onset of diabetic nephropathy. Diabetes : 225–233. 16380497

[40] Lodha S, Dani D, Mehta R, Bhaskaran M, Reddy K, et al (2002) Angiotensin II-induced mesangial cell apoptosis: role of oxidative stress. Mol Med 8:830–840. 12606818 PMC2039960

[41] Liu Y (2009) Advanced oxidation protein products: a causative link between oxidative stress and podocyte depletion. Kidney Int 76:1125–1127. 19910947 10.1038/ki.2009.352

[42] Morais C, Westhuyzen J, Metharom P, Healy H (2005) High molecular weight plasma proteins induce apoptosis and Fas/FasL expression in human proximal tubular cells. Nephrol Dial Transplant 20:50–58. 15522900 10.1093/ndt/gfh561

[43] Tsuda H, Kawada N, Kaimori JY, Kitamura H, Moriyama T, et al (2012) Febuxostat suppressed renal ischemia-reperfusion injury via reduced oxidative stress. Biochem Biophys Res Commun 427:266–272. 22995295 10.1016/j.bbrc.2012.09.032

[44] Hansell P, Welch WJ, Blantz RC, Palm F (2013) Determinants of kidney oxygen consumption and their relationship to tissue oxygen tension in diabetes and hypertension. Clin Exp Pharmacol Physiol 40:123–137. 23181475 10.1111/1440-1681.12034 PMC3951849

[45] Sanchez-Niño MD, Benito-Martin A, Ortiz A (2010) New paradigms in cell death in human diabetic nephropathy. Kidney Int 78:737–744. 20703212 10.1038/ki.2010.270

[46] Devaraux QL (1998) IAPs block apoptotic events induced by Caspase 8 and cytochrome c by direct inhibition of distinct caspases. EMBO J 17:2215–2223. 9545235 10.1093/emboj/17.8.2215 PMC1170566

[47] Schimmer AD, Dalili S, Batey RA, Riedl SJ (2006) Targeting XIAP for the treatment of malignancy. Cell Death Differ 13:179–188. 16322751 10.1038/sj.cdd.4401826

[48] Han HJ, Lim MJ, Lee YJ, Lee JH, Yang IS, et al (2007) Uric acid inhibits renal proximal tubule cell proliferation via at least two signaling pathways involving PKC, MAPK, cPLA2, and NF-kappaB. Am J Physiol Renal Physiol 292:F373–381. 16985215 10.1152/ajprenal.00104.2006

[49] Bose B, Badve SV, Hiremath SS, Boudville N, Brown FG, et al (2014) Effects of uric acid-lowering therapy on renal outcomes: a systematic review and meta-analysis. Nephrol Dial Transplant 29:406–413. 24042021 10.1093/ndt/gft378

[50] Sánchez-Lozada LG, Tapia E, Soto V, Avila-Casado C, Franco M, et al (2008) Treatment with the xanthine oxidase inhibitor febuxostat lowers uric acid and alleviates systemic and glomerular hypertension in experimental hyperuricaemia. Nephrol Dial Transplant 23:1179–1185. 18048425 10.1093/ndt/gfm783

[51] Høieggen A, Alderman MH, Kjeldsen SE, Julius S, Devereux RB, et al (2004)The impact of serum uric acid on cardiovascular outcomes in the LIFE study. Kidney Int 65:1041–1049. 14871425 10.1111/j.1523-1755.2004.00484.x

[52] Miao Y, Ottenbros SA, Laverman GD, Brenner BM, Cooper ME, et al (2011) Effect of a reduction in uric acid on renal outcomes during losartan treatment: a post hoc analysis of the reduction of endpoints in non-insulin-dependent diabetes mellitus with the Angiotensin II Antagonist Losartan Trial. Hypertension 58:2–7. 21632472 10.1161/HYPERTENSIONAHA.111.171488

[53] Smink PA, Bakker SJ, Laverman GD, Berl T, Cooper ME, et al (2012) An initial reduction in serum uric acid during angiotensin receptor blocker treatment is associated with cardiovascular protection: a post-hoc analysis of the RENAAL and IDNT trials. J Hypertens 30:1022–1028. 22388234 10.1097/HJH.0b013e32835200f9

